# Diabetes drugs for nonalcoholic fatty liver disease: a systematic review

**DOI:** 10.1186/s13643-019-1200-8

**Published:** 2019-11-29

**Authors:** Ian Blazina, Shelley Selph

**Affiliations:** Pacific Northwest Evidence-based Practice Center, Oregon Health & Science University, 3181 SW Sam Jackson Park Rd, Mailcode: BICC, Portland, OR 97239 USA

**Keywords:** Systematic review, Nonalcoholic fatty liver disease, Diabetes, Pharmacotherapy

## Abstract

**Background:**

Fatty liver is associated with obesity, type 2 diabetes, hyperlipidemia, hypertension, and metabolic syndrome. While there are no approved drugs for the treatment of nonalcoholic fatty liver disease (NAFLD) or nonalcoholic steatohepatitis, strategies to ameliorate fatty liver often target these related diseases. We sought to determine if any medications approved by the US Food and Drug Administration to treat diabetes are helpful in reducing weight and improving steatohepatitis in patients with NAFLD.

**Methods:**

We conducted a systematic review of published and unpublished studies evaluating the comparative effectiveness and harms of diabetes medications for the treatment of NAFLD. We searched MEDLINE, EMBASE, Cochrane Database of Systematic Reviews, and the Cochrane Central Register of Controlled Trials through 3rd quarter, 2019 using terms for included drugs and indications.

**Results:**

We screened 1591 citations and included 18 trials of diabetes drugs to treat NAFLD. Studies of metformin found no difference from placebo in steatosis, fibrosis, NAFLD activity score, or resolution of NASH. While weight and glucose control were improved with metformin, it did not substantially impact liver disease. Studies of pioglitazone in NASH patients found benefits in liver function, liver fat, and NASH resolution, though significant increases in weight may be cause for concern. Evidence for other thiazolinediones was more limited and had somewhat mixed results, but findings were generally consistent with those for pioglitazone: liver fat and function and glucose measures improved, but weight also increased. We found some evidence that liraglutide improves liver fat, liver function, and HbA1c and is effective at resolving NASH and reducing weight. Exenatide performed less well but also resulted in significant reductions in liver fat and weight.

**Conclusions:**

Consistent with existing clinical practice guidelines, which recommend lifestyle intervention and treatment for comorbidities related to fatty liver disease as first-line treatment, trial evidence supports the efficacy of some diabetes drugs (especially pioglitazone) in patients with NAFLD or NASH, though weight gain with some diabetes drugs may warrant caution. Larger trials are needed to better characterize the efficacy and harms of diabetes pharmacotherapy in these patients.

## Background

Nonalcoholic fatty liver disease (NAFLD), the accumulation of excess fat in the liver (steatosis) not resulting from excessive alcohol consumption or another secondary cause, is a growing public health issue associated with the global epidemics of obesity and type 2 diabetes [1]. NAFLD represents a spectrum of diseases, from mild steatosis to nonalcoholic steatohepatitis (NASH) and cirrhosis. The prevalence of NAFLD in North America is estimated to be 20% to 30%, with around 2 to 3% of the population having NASH [2]. NAFLD is a common cause of cirrhosis, end-stage chronic liver disease, liver transplantation, and hepatocellular carcinoma [3]; liver-related mortality is about twice as high among those with NAFLD than those without [2].

Fatty liver is associated with obesity, type 2 diabetes, hyperlipidemia, hypertension, and metabolic syndrome. While there are no approved drugs for the treatment of NAFLD or NASH, strategies to ameliorate fatty liver often target these related diseases [4].

We performed a systematic review to determine if any medications approved by the US Food and Drug Administration (FDA) to treat diabetes are helpful in reducing weight and improving steatohepatitis in patients with NAFLD in the setting of many new drugs in development seeking an indication for NAFLD or NASH. The original review, which was commissioned by the Drug Effectiveness Review Project and is not publicly available, also evaluated the success of weight loss drugs, dyslipidemia drugs, diet, and exercise in weight loss and improvement of NAFLD. Here, we focus on the evidence for diabetes medications, which was the most well-studied intervention area.

We sought evidence to answer the following questions:

1. What is the comparative efficacy and effectiveness of FDA-approved drugs that are used off-label to treat nonalcoholic fatty liver disease?
2. What are the comparative harms of FDA-approved drugs that are used off-label to treat nonalcoholic fatty liver disease?

## Methods

We followed systematic review methodology and procedures developed specifically for the Drug Effectiveness Review Project (DERP) [5] and that are in accordance with current guidance for systematic reviews.

### Data sources and searches

We searched MEDLINE, EMBASE, Cochrane Database of Systematic Reviews, and the Cochrane Central Register of Controlled Trials through 3rd quarter, 2019, using terms for included drugs and indications (Additional file 1). We consulted medical reviews from the Food and Drug Administration’s Center for Drug Evaluation and Research and requested additional unpublished trial data from relevant pharmaceutical companies.

### Study selection

Eligible studies were head-to-head or placebo-controlled randomized controlled studies of adults with nonalcoholic fatty liver disease (including NASH) who received an FDA-approved diabetes drug to treat NASH/NAFLD. We excluded any drug developed specifically for the treatment of NAFLD, including the FDA-approved obeticholic acid as well as drugs in development specifically for treatment of NALFD (selonsertib, elafibranor, and cenicriviroc), as well as studies that only evaluated different doses of the same drug (dose-ranging studies). Benefit outcomes of interest included changes in alanine aminotransferase test (ALT), aspartate aminotransferase test (AST), liver fat, liver fibrosis, and resolution of NAFLD; weight loss (e.g., pounds lost, change in BMI, loss of 10% body weight); long-term health outcomes (e.g., mortality, need for liver transplant); and HbA1c or other glucose outcomes. Relevant harms included serious adverse events and withdrawals due to adverse events. We required studies to have at least 30 patients per treatment arms of interest unless the trial included liver histology, the gold standard, at the conclusion of the trial; in which case, we reduced the required sample size to 20 per treatment arm.

One reviewer screened citations and a second reviewer assessed excluded citations. Two reviewers independently evaluated full-text articles by applying the inclusion criteria and resolved disagreements by consensus.

### Data abstraction and quality assessment

Information on population characteristics, interventions, subject enrollment and discontinuation, and results for effectiveness and harm outcomes were abstract by one reviewer. The second reviewer verified abstracted data.

Study quality was assessed independently by two reviewers according to the DERP’s methods [5], focusing on methods of randomization, allocation concealment, blinding of providers, outcome assessors, and patients; similarity of group characteristics at baseline, especially of prognostic factors; attrition rate; and the use of intent-to-treat analysis. Studies that met all criteria were rated as good quality; studies with an element at high risk of bias or failed to meet combinations of criteria were rated as poor quality; and the remaining studies were rated fair quality. Disagreements between reviewers were resolved by consensus.

### Grading strength of evidence

We graded strength of evidence according to the Agency for Healthcare Research and Quality’s (AHRQ) guidance for the Evidence-based Practice Center Program [6]. Similar to the GRADE method, this approach assesses risk of bias, consistency, directness, and precision of the evidence. Strength of evidence was graded for key outcome measures of liver fat, weight change, and ALT or AST elevations. Grades reflect the strength of the body of evidence to answer key questions on the effectiveness and harms of included drugs, not general efficacy of the drugs. Two reviewers independently assessed each domain for each outcome and differences were resolved by consensus.

## Results

**Fig. 1:**
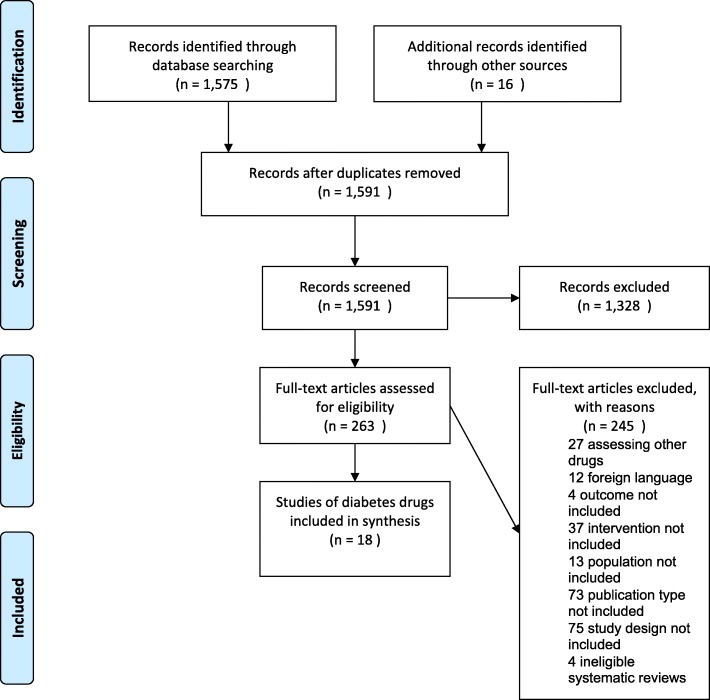
PRISMA 2009 Flow Diagram

We screened 1591 citations and included 39 trials (in 41 publications) in the primary report; here, we report only the evidence pertaining to diabetes drugs (18 trials in 17 publications) [7–22] (Fig. 1). Most trials were small (*N* < 100 per treatment arm) and rated fair quality, primarily due to unclear blinding, unclear allocation concealment, and high attrition. Five trials were rated good quality [7, 9, 18, 19, 22]. Some trials enrolled patients at the more severe end of NAFLD continuum who were diagnosed with NASH, and some trials required enrollees to also have metabolic syndrome, prediabetes, type 2 diabetes, hypertension, and/or be overweight or obese.

Twelve individual randomized trials in NAFLD patients with [7, 8] and without [9–17, 22, 23] type 2 diabetes included treatment with a diabetes drug in 1 or more treatment arms. In addition, 3 trials enrolled patients with prediabetes or type 2 diabetes [18–20]. We [23] also included an individual patient data (IPD) meta-analysis of 6 RCTs of liraglutide in patients with diabetes [21]. Most studies did not report harm outcomes. Ten studies evaluated thiazolidinediones (Table 1), three studies evaluated GLP-1 agonists (Table 2), six studies evaluated metformin (Table 3), and one study evaluated a DPP-4 inhibitor (Table 4).

**Table 1:** Studies of thiazolidinediones to treat nonalcoholic fatty liver disease

Author, year country trial name (quality rating)	Population demographics	Interventions (group sizes) duration	Efficacy/effectiveness outcomes A vs. B	Harms A vs. B
Aithal, 2008 [12] UK (fair)	Nondiabetic adults with biopsy-confirmed NASH Age: 53 y % female: 39 Ethnicity: NR BMI, kg/m^2^: 30.3	A: Pioglitazone 30 mg/d (*n* = 37) B: Placebo (*n* = 37) Duration: 12 months	Number (%) with improvement (*P* value), between-groups *P* value: Fibrosis: 9/31 (29%) (*P* = 0.006) vs. 6/30 (20%) (*P* = 0.81), *P* = 0.05 Steatosis: 15/31 (48%) (*P* = 0.001) vs. 11/30 (37%) (*P* = 0.03), *P* = 0.19 Changes from baseline (*P* value), between-groups *P* value: Weight, kg: 2.6 (*P* = 0.005) vs. − 3.5 (*P* = 0.69), *P* = 0.02 ALT: − 37.7 (*P* = 0.02) vs. − 6.9 (*P* = 0.41), *P* = 0.009	Serious AEs: NR Withdrawal due to AEs: 3/37 (8.1%) vs. 4/37 (10.8%)
Anushiravani, 2019 [22] Iran (good)	Adults with probable NAFLD with or without elevated ALT/AST Age: 47 y % female: 49 Ethnicity: NR BMI, kg/m^2^: 25.1 vs. 26.1	A. Pioglitazone 15 mg/d (*n* = 30) B. Placebo (*n* = 30) Duration: 3 months	Changes from baseline (*P* value), between-groups *P* value: BMI: − 0.6 vs. − 0.7 kg/m^2^; *P* = NS ALT: − 8.6 vs. − 0.6; *P* < 0.001 AST: − 6.7 vs. − 0.9; *P* < 0.001	None
Belfort, 2006 [19] US (good)	Adults with type 2 diabetes or impaired glucose tolerance and biopsy-confirmed NASH Age: 51 y % female: 55 Ethnicity: NR BMI, kg/m^2^: 33.2	A: Pioglitazone 30 mg/d for 2 months, then 45 mg/d (*n* = 29) B: Placebo (*n* = 25) Duration: 6 months	Percent with fibrosis improvement: 46% vs. 33%, *P* = 0.08 Changes from baseline (*P* value), between-groups *P* value: AST: − 19 (*P* < 0.001) vs. − 9 (*P* = 0.08), *P* = 0.04 ALT: − 39 (*P* < 0.001) vs. − 21 (*P* = 0.033), *P* < 0.001 Weight, kg: 2.5 (*P* < 0.001) vs. − 0.5 (*P* = 0.53), *P* = 0.003 BMI: 1.1 (*P* < 0.001) vs. − 0.2 (*P* = 0.62), *P* = 0.005	Serious AEs: NR Withdrawal due to AEs: 1/29 (3.5%) vs. 1/25 (4.0%)
Cusi, 2016 [18] US (good)	Patients with prediabetes or type 2 diabetes and nonalcoholic steatohepatitis proven by biopsy Age: 50.5 Sex: 70.3 % male Ethnicity: 24.8% White, 67.3% Hispanic, 0.08% Other BMI: 34.4 Mean NAS: 4.5 Mean fibrosis stage: 1.0 HbA1C with diabetes: 6.95%, without diabetes 5.7% Participants with diagnosed NASH: 86.1% Mean ALT: 59.5	A. Pioglitazone 45 mg per day (*n* = 50) B. Placebo, (*n* = 51) All patients were prescribed a hypocaloric diet. Both groups followed with open-label phase with Pioglitazone for 18 months Duration: 18 months	Greater than 2 point reduction of NAS without worsening fibrosis: 29% vs. 17%, *P* < 0.001 Fibrosis; greater than 1 point improvement: 39% vs. 25%, *P* > 0.05 Fibrosis mean change in score improved with pioglitazone: 0 vs. − 0.5, *P* < 0.05 Weight: pioglitazone group gained 2.5 kg, *P* < 0.05 BMI: treatment group increase of 2.5 kg, *P* < 0.05	NR
Rana, 2016 [10] India (fair)	Patients with ultrasound diagnosed NAFLD without history of use of insulin sensitizers or hypolipidemic drug use Age: NR Sex: NR Ethnicity: Indian Liver status: AST 55.14 IU/mL; ALT 64.30 (AST and ALT were different at baseline between treatment groups) BMI: 27.95	A. Metformin (31) B. Rosuvastatin (34) C. Pioglitazone (33) Duration: 24 weeks	Change in ultrasound score (fatty liver) at 24 weeks: our analysis A vs. C: 0.065 vs. − 0.697 (*P* < 0.001) B vs. C: − 1.265 vs. − 0.697 (*P* = 0.008) Weight change at 24 weeks: our analysis A vs. C: − 4.76 vs. 0.03 (*P* < 0.001) B vs. C: − 4.25 vs. 0.03 (*P* < 0.001) AST change at 24 weeks: our analysis A vs. C: − 14.07 vs. − 23.73 (*P* = 0.04) B vs. C: 8.35 vs. − 23.73 (*P* < 0.001) ALT change at 24 weeks: our analysis A vs. C: − 15.55 vs. − 24.67 (*P* = 0.13) B vs. C: 8.06 vs. − 24.67 (*P* < 0.001)	NR
Razavizade, 2013 [14] Iran (fair)	Adults with NAFLD assessed via ultrasonography and predictive formula Age: 35.3 y % female: 15 Ethnicity: NR BMI, kg/m^2^: 27.7 Diabetes: 7.5%	A: Metformin 1000 mg/d (*n* = 40) B: Pioglitazone 30 mg/d (*n* = 40) Duration: 4 months	Changes from baseline (*P* value), between-groups *P* value: Liver fat fraction: − 2.53 (*P* < 0.01) vs. − 3.23 (*P* < 0.01), *P* = 0.48 AST: − 10.83 (*P* < 0.01) vs. − 13.75 (*P* < 0.01), *P* = 0.56 ALT: − 21.75 (*P* < 0.01) vs. − 37.53 (*P* < 0.01), *P* = 0.07 Weight, kg: − 2.73 (*P* < 0.01) vs. − 1.18 (*P* = 0.04), *P* = 0.05	Serious AEs: NR Withdrawal due to AEs: none
Sanyal, 2010 [13] US PIVENS (fair)	Nondiabetic adults with biopsy-confirmed NASH Age: 46.3 y % female: 60 Ethnicity, % white: 88 BMI, kg/m^2^: 34	A: Pioglitazone 30 mg/d (*n* = 80) B: Vitamin E 800 IU/d (*n* = 84) C: Placebo (*n* = 83)	Changes from baseline (*P* value vs. placebo): NASH improvement, *n* (%): 27/80 (34%) (*P* = 0.04) vs. 36/84 (43%) (*P* = 0.001) vs. 16/83 (19%) NAFLD activity score: − 1.9 (*P* < 0.001) vs. − 1.9 (*P* < 0.001) vs. − 0.5 Steatosis: − 0.8 (*P* < 0.001) vs. − 0.7 (*P* < 0.001) vs. − 0.1 Fibrosis: − 0.4 (*P* = 0.10) vs. − 0.3 (*P* = 0.19) vs. − 0.1 AST: − 20.4 (*P* < 0.001) vs. − 21.3 (*P* < 0.001) vs. − 3.8 ALT: − 40.8 (*P* < 0.001) vs. − 37.0 (*P* = 0.001) vs. − 20.1 Weight, kg: 4.7 (*P* < 0.001) vs. 0.4 (*P* = 0.65) vs. 0.7	Serious AEs: NR Withdrawal due to AEs: None
Sharma, 2012 [11] India (fair)	Adults with biopsy-confirmed NASH Age: 38.9 y % female: 46 Ethnicity: NR BMI, kg/m^2^: 24.9 Diabetes: NR	A: Pentoxifylline 1200 mg/d (*n* = 29) B: Pioglitazone 30 mg/d (*n* = 30) Duration: 24 weeks	Changes from baseline (*P* value), between-groups *P* value: Brunt score: − 0.34 (*P* = 0.10) vs. − 1.2 (*P* = 0.005), *P* = 0.04 Steatosis: − 0.83 (*P* = 0.02) vs. − 1.18 (*P* = 0.005), *P* = 0.60 Fibrosis: 0.08 (*P* = 0.70) vs. − 0.46 (*P* = 0.19), *P* = 0.26	Serious AEs: NR Withdrawal due to AEs: None
Ratziu, 2008 [17] France FLIRT (fair)	Adults with biopsy-confirmed NASH Age: 53.6 % female: 41 Ethnicity: NR BMI, kg/m^2^: 31 Diabetes: 32%	A: Rosiglitazone 8 mg/d (4 mg/d for first month) (*n* = 32) B: Placebo (*n* = 31) Duration: 12 months	Changes from baseline, between-groups *P* value: NAFLD activity score: − 1 vs. 0, *P* = 0.60 Steatosis, % reduction: − 20% vs. − 5%, *P* = 0.02 Fibrosis: 0.03 vs. − 0.18, *P* = 0.43 ALT, number (%) achieving normalization: 12/32 (38%) vs. 2/31 (7%), *P* = 0.005 ALT, mean % change from baseline: − 28% vs. − 2%; mean reduction, − 44% vs. 0% AST, mean % change from baseline: − 8% vs. 9%; mean reduction, − 62% vs. + 15%	Serious AEs: NR Withdrawal due to AEs: 1/32 (3.1%) vs. 0/31 Dose reduction due to AEs: 5/32 (15.6%) vs. 1/31 (3.2%)
Torres, 2011 [16] US (fair)	Adults with biopsy-confirmed NASH Age: 49.4 y % female: 36 Ethnicity, %: Caucasian: 65 Hispanic: 22 BMI, kg/m^2^: 33.2 Diabetes: 16.7%	A: Rosiglitazone 8 mg/d (*n* = 50) B: Rosiglitazone 8 mg/d + metformin 1000 mg/d (*n* = 50) C: Rosiglitazone 8 mg/d + losartan 50 mg/d (*n* = 50) Duration: 48 weeks	Subjects with final biopsy: 26 vs. 28 vs. 35 Changes from baseline, between-groups *P* value: Resolution of definite NASH, *n* (%): 12/26 (46%) vs. 10/28 (36%) vs. 10/35 (29%), NR NAFLD activity score: − 1.77 vs. − 1.32 vs. − 1.37, *P* = 0.671 Steatosis: − 0.85 vs. − 0.82 vs. − 0.74, *P* = 0.905 Fibrosis: − 0.70 vs. − 0.59 vs. − 0.32, *P* = 0.302 AST: − 39.6 vs. − 35.0 vs. − 48.7, NS (exact *P* value NR) ALT: − 17.4 vs. − 19.9 vs. − 21.7, NS (exact *P* value NR) Weight, kg: 0.9 vs. − 1.2 vs. 3.7, *P* = 0.051	Serious AEs: NR Withdrawal due to AEs: not reported by group

**Table 2:** Studies of GLP-1 agonists to treat nonalcoholic fatty liver disease

Author, year country trial name (quality rating)	Population demographics	Interventions (group sizes) duration	Efficacy/effectiveness outcomes A vs. B	Harms A vs. B
Armstrong, 2013 [21] Multinational LEAD & LEAD-2 (fair)	Patients with type 2 diabetes who were unable to maintain glycemic control (HbA1c ≥ 7%) with diet and exercise alone, or with oral antidiabetic treatment Age: 55.9 years Gender, % Female: 46.5 Ethnicity, %: White: 78.6 Asian/Hawaiian/Pacific Islander: 12.7 Black/African American: 5.8 Other: 2.1 BMI: NR NAFLD/NASH Stage: NR Liver enzymes (IU/L): Total ALT mean (SD): 29.4 (16.6) Normal ALT mean (SD): 19.1 (5.6) Abnormal ALT mean (SD): 39.4 (17.7)	LEAD A: Liraglutide 0.6 mg/day (475) B: Liraglutide 1.2 mg/day (896) C: Liraglutide 1.8 mg/day (1363) D: Placebo (524) Duration: 26 weeks LEAD-2 0.6, 1.2, or 1.8 mg/day liraglutide, 4 mg/day glimepiride or placebo, all in combination with metformin	LEAD In patients with elevated ALT, liraglutide 1.8 significantly reduced ALT compared with placebo (and was dose responsive); however, after correcting for change in weight, the difference was no longer significant: Mean difference − 1.41, *P* = 0.21, with similar finding after correcting for reduction in HbA1c: Mean difference 0.57, *P* = 0.63. Lower doses of liraglutide had similar effects as placebo LEAD2 64% of patients had elevated liver fat on CT; as above liraglutide improved liver fat in a dose dependent way; however, there was no significant differences between liraglutide and placebo after correcting either for weight loss or HbA1c (*P* = 0.90 and 0.73, respectively)	LEAD WAE: 9% vs. 9% vs. 3% (liraglutide vs. placebo, *P* < 0.001) SAE: 7% vs. 6% vs. 6% GI disorders: 46% vs. 45% vs. 18% (liraglutide vs. placebo, *P* < 0.001) LEAD2 NR
Armstrong, 2016 [9] UK LEAN (good)	Patients had histologically confirmed NASH Age: 51 Sex: 60% male Ethnicity: White: 88% Asian: 4% Black: 2% Other: 6% Liver status: NAS: 4.9; ALT: 72 IU/mL; F3-F4: 52% BMI: 36 Diabetes: 33%	A. Liraglutide 1.8 mg (26) B. Placebo (26) Duration: 48 weeks	Resolution of NASH: 39% vs. 9% (RR 4.3, 95% CI 1.0 to 17.7) Change in NAS: − 1.3 vs. − 0.8, *P* = 0.24 Change in fibrosis stage: − 0.2 vs. 0.2, *P* = 0.11 Patients with improvement in fibrosis: 26% vs. 14%, *P* = 0.46 Patient with worsening fibrosis: 9% vs. 36%, *P* = 0.04 Change in ALT: − 26.6 vs. − 10.2, *P* = 0.16 Change in AST: − 27 vs. + 9 IU/L; *P* = 0.025	WAE: 8% vs. 4% (*P* = 0.56) SAE: 8% vs. 8% GI disorders: 81% vs. 65% (*P* = 0.27)
Shao, 2014 [8] China (fair)	Patients with type 2 diabetes, obesity, NAFLD, and elevated liver enzymes with normal renal function Age: 43 Sex: 48% male Ethnicity: Chinese Mild NAFLD: 40% Moderate NAFLD: 42% Severe: 18% BMI: 30 HbA1c: 7.64%	A. exenatide + glargine (30) B. Intensive insulin: Insulin aspart + insulin glargine (30) Duration: 12 weeks	Reversal rate of NAFLD based on ultrasound: A vs. B: 93% vs. 67% , *P* < 0.01 Differences in weight change post minus pretreatment: A vs. B: − 7.77 kg vs. 3.27, *P* < 0.001 No difference between groups in change in HbA1c: A vs. B: − 1.42% vs. − 1.31%, *P* > 0.05	NR

**Table 3:** Studies of metformin to treat nonalcoholic fatty liver disease

Author, year country trial name (quality rating)	Population demographics	Interventions (group sizes) duration	Efficacy/effectiveness outcomes	Harms
			A vs. B	A vs. B
Anushiravani, 2019 [22] Iran (good)	Adults with probable NAFLD with or without elevated ALT/AST Age: 47 y % female: 49 Ethnicity: NR BMI, kg/m^2^: 25.1 vs. 26.1	A. Metformin 500 mg/d (*n* = 30) B. Placebo (*n* = 30) Duration: 3 months	Changes from baseline (*P* value), between-groups *P* value: BMI: − 0.6 vs. − 0.7 kg/m^2^; *P* = NS ALT: − 10.1 vs. − 0.6; *P* < 0.001 AST: − 6.4 vs. − 0.9; *P* < 0.001	None
Haukeland, 2009 [15] Norway (fair)	Adults with biopsy-confirmed NAFLD Age: 47.4 y % female: 27.2 Ethnicity, % white: 86.4% BMI, kg/m^2^: 30.8 Diabetes: 27.3%	A: Metformin 2500 mg/d (3000 mg if weight > 90 kg) (*n* = 24) B: Placebo (*n* = 24) Duration: 6 months	Percentage with improvement (*P* value change from baseline); between-groups *P* value: Steatosis: 25% (*P* = 0.10) vs. 38% (*P* = 0.03); *P* = 0.052 Fibrosis: 5% (*P* = 1.00) vs. 17% (*P* = 0.17); *P* = 0.36 NAFLD activity score: 20% (*P* = 0.23) vs. 50% (*P* = 0.12); *P* = 0.06 Changes from baseline (*P* value); between-groups *P* value: Weight, kg: − 4.3 (*P* < 0.001) vs. 0.3 (*P* = 0.45); *P* < 0.001 BMI: 1.3 (*P* < 0.001) vs. 0.1 (*P* = 0.59); *P* < 0.001	Serious AEs: NR Withdrawal due to AEs: 2/24 (8.3%) vs. 0/24 (0%)
Omer, 2010 [20] Turkey (fair)	Adults with type 2 diabetes or impaired glucose tolerance and biopsy-confirmed NAFLD Age: 48.9 y % female: 45.3 Ethnicity: NR BMI, kg/m^2^: 30.6 Diabetes: NR	A: Metformin 1700 mg/d + rosiglitazone 4 mg/d (*n* = 22) B: Metformin 1700 mg/d (*n* = 22) C: Rosiglitazone 4 mg/d (*n* = 20) Duration: 12 months	Changes from baseline (*P* value): NAFLD score (*n* = 10–13): − 3.9 (*P* = 0.026) vs. 0.7 (*P* = 0.726) vs. − 2.6 (*P* = 0.012) AST: − 15.4 (*P* = 0.01) vs. − 13.0 (*P* = NS) vs. − 13.2 (*P* = 0.005) ALT: − 22.7 (*P* = 0.017) vs. − 16.7 (*P* = NS) vs. − 36.2 (*P* < 0.0001) BMI: − 1.3 (*P* = 0.006) vs. − 3.2 (*P* = 0.002) vs. − 0.3 (*P* = NS)	Serious AEs: NR Withdrawal due to AEs: Not adequately reported
Rana, 2016 [10] India (fair)	Patients with ultrasound diagnosed NAFLD without history of use of insulin sensitizers or hypolipidemic drug use Age: NR Sex: NR Ethnicity: Indian Liver status: AST 55.14 IU/mL; ALT 64.30 (AST and ALT were different at baseline between treatment groups) BMI: 27.95	A. Metformin (31) B. Rosuvastatin (34) C. Pioglitazone (33) Duration: 24 weeks	Change in ultrasound score (fatty liver) at 24 weeks: our analysis A vs. B: 0.065 vs. − 1.265 (*P* < 0.001) A vs. C: 0.065 vs. − 0.697 (*P* < 0.001) Weight change at 24 weeks: our analysis A vs. B: − 4.76 vs. − 4.25 (*P* = 0.13) A vs. C: − 4.76 vs. 0.03 (*P* < 0.001) AST change at 24 weeks: our analysis A vs. B: − 14.07 vs. 8.35 (*P* < 0.001) A vs. C: − 14.07 vs. − 23.73 (*P* = 0.04) ALT change at 24 weeks: our analysis A vs. B: − 15.55 vs. 8.06 (*P* < 0.001) A vs. C: − 15.55 vs. − 24.67 (*P* = 0.13)	NR
Razavizade, 2013 [14] Iran (fair)	Adults with NAFLD assessed via ultrasonography and predictive formula Age: 35.3 y % female: 15 Ethnicity: NR BMI, kg/m^2^: 27.7 Diabetes: 7.5%	A: Metformin 1000 mg/d (*n* = 40) B: Pioglitazone 30 mg/d (*n* = 40) Duration: 4 months	Changes from baseline (*P* value), between-groups *P* value: Liver fat fraction: − 2.53 (*P* < 0.01) vs. − 3.23 (*P* < 0.01), *P* = 0.48 AST: − 10.83 (*P* < 0.01) vs. − 13.75 (*P* < 0.01), *P* = 0.56 ALT: − 21.75 (*P* < 0.01) vs. − 37.53 (*P* < 0.01), *P* = 0.07 Weight, kg: − 2.73 (*P* < 0.01) vs. − 1.18 (*P* = 0.04), *P* = 0.05	Serious AEs: NR Withdrawal due to AEs: None.

**Table 4:** Studies of DPP-4 inhibitors to treat nonalcoholic fatty liver disease

Author, year country trial name (quality rating)	Population demographics	Interventions (group sizes) duration	Efficacy/effectiveness outcomes A vs. B	Harms A vs. B
Deng, 2017 [7] China (good)	Patients with type 2 diabetes for less than 2 years without complications and fatty liver diagnosed by ultrasound Age: 64 Sex: 75% male Ethnicity: Liver status: ALT 35 IU/mL; AST: 32 IU/mL BMI: 24 Diabetes: HbA1c 7.4%	A. Sitagliptin 50 to 100 mg (36) B. Diet and exercise Duration: 52 weeks	No difference in change in AST (*P* = 0.99) or ALT (*P* = 0.97) between treatment with sitagliptin vs. diet and exercise Greater decrease in HbA1c with sitagliptin (− 0.81) vs. diet and exercise (− 0.25), *P* < 0.01 at 52 weeks (also at 13, 26, and 39 weeks)	NR

### Patients with NAFLD and diabetes

#### Liraglutide

An IPD meta-analysis of 6 RCTs (*N* = 3258) [21] and 2 additional, unique trials [7, 8] (*N* = 132) enrolled NAFLD patients with type 2 diabetes. All RCTs in the IPD meta-analysis were 26-week trials and treated patients with liraglutide 0.6 mg, liraglutide 1.2 mg, liraglutide 1.8 mg, or placebo. The IPD meta-analysis found that in patients with an elevated ALT (*N* = 1387), liraglutide 1.8 mg reduced ALT to a significantly greater degree than placebo, but the effect was lost after correcting for patient weight loss (*P* = 0.21) or after correcting for improvement in HbA1c (*P* = 0.63). Findings were similar for decrease in hepatic fat. In a sub-study (*n* = 149), there was a trend for reduction in liver fat (measured with CT scanning) with liraglutide 1.8 mg (*P* = 0.07), but the effect was lost after adjusting for weight loss (*P* = 0.90) or HbA1c (*P* = 0.73). While the incidence of serious adverse events was similar between liraglutide and placebo (6.5% vs. 6%, RR 1.10, 95% CI 0.75 to 1.62), study withdrawal due to adverse events and gastrointestinal disorder was significantly more likely in patients treated with liraglutide (withdrawal due to adverse events: 9% vs. 3%, RR 3.59, 95% CI 2.07 to 6.24; GI disorders 45% vs. 18%, RR 2.54, 95% CI 2.10 to 3.08).

#### Exenatide

A trial of exenatide (*N* = 132) conducted in China enrolled patients with NAFLD and diabetes and reported efficacy outcomes only [8]. Twelve weeks of exenatide treatment (5 mg twice daily for 4 weeks then 10 mg twice daily for 8 weeks) was associated with greater reversal of liver fat assessed by ultrasound than intensive insulin therapy (93% vs. 67%, *P* < 0.01) [8]. At the conclusion of therapy, 40 to 43% of patients no longer had a fatty liver, and in the exenatide group, 0 out of 6 patients (0%) still had severe disease compared with 3 out of 5 patients (60%) in the insulin group (*P* = 0.03, our analysis). Body weight was also significantly reduced with exenatide compared with intensive insulin (− 7.77 kg vs. + 3.27 kg, *P* < 0.001) but there was no differential treatment effect on HbA1c (− 1.42% vs. − 1.31%, *P* > 0.05).

#### Sitagliptin

A small RCT conducted in China found no difference between sitagliptin 50 to 100 mg compared with diet and exercise on the liver function tests AST and ALT after 1 year of treatment, although sitagliptin treatment was associated with greater reduction in HbA1c (− 0.81 vs. − 0.25, *P* < 0.01) [7].

#### Pioglitazone

Two RCTs enrolled patients with histologically confirmed NASH and prediabetes or diabetes as determined by an abnormal glucose tolerance test [18, 19]. In the first trial, all 101 patients were advised to follow a hypocaloric diet (500 kcal/d deficit) and were then randomized to pioglitazone 45 mg or placebo for 18 months (some patients were also taking metformin, a sulfonylurea, and/or insulin) [18]. A greater than or equal to 2-point reduction in Nonalcoholic fatty liver disease Activity Score (NAS) without worsening of fibrosis was the primary outcome and favored pioglitazone (58% vs. 18%, RR 3.28, 95% CI 1.74 to 6.22, our analysis). Resolution of NASH based on liver histology also favored pioglitazone (52% vs. 20%, RR 2.65, 95% CI 1.43 to 4.91, our analyses). HbA1c improved slightly with pioglitazone compared with placebo (− 0.6%, *P* = 0.009); however, treatment with pioglitazone was also associated with significant gain in weight (2.5 kg, *P* = 0.02).

In the second trial, 55 patients were counseled to follow a hypocaloric diet and then randomized to treatment with pioglitazone 45 mg or placebo for 6 months [19]. Pioglitazone treatment versus placebo was associated with greater improvements in HbA1c (− 0.7% vs. − 0.1%, *P* = 0.008), AST (− 19 vs. − 9 IU/L, *P* = 0.04), and ALT (− 39 vs. − 21 IU/L, *P* < 0.001). The proportion of participants who experienced improvement in hepatic fat was greater in the pioglitazone group (65% vs. 38%, *P* = 0.003), while there were no differences between treatments in fibrosis score. Pioglitazone treatment was also associated with weight gain compared with placebo (+ 2.5 kg vs. − 0.5 kg, *P* = 0.003). Study withdrawals due to adverse events were few and not different between groups.

#### Rosiglitazone and metformin

One trial randomized 64 patients with NAFLD to treatment with rosiglitazone 4 mg/day, metformin 1700 mg/day, or combination therapy for 12 months [20]. All patients also had impaired glucose metabolism (type 2 DM or impaired glucose tolerance) and elevated liver transaminases and were on a diet and exercise program for 12 weeks prior to the start of the trial. Baseline insulin levels were significantly different between groups (10.1 mg/dL in the rosiglitazone group, 14.9 mg/dL in the metformin group, 16.6 mg/dL in the combined therapy group, *P* = 0.04) but not significantly different between groups in other baseline characteristics. The trial reported decreased BMI from baseline for 12 months for metformin (30.8 to 27.6 kg/m^2^, *P* = 0.002) and for rosiglitazone plus metformin (32.5 to 31.2 kg/m^2^, *P* = 0.006). However, we believe that the values for rosiglitazone plus metformin to be in error as the correlation between baseline and 12 months would have to be greater than 0.96, which is extremely high and not likely correct. Postprandial glucose was decreased in all groups. Post-treatment liver biopsy was performed on 55% of patients and NAFLD activity score favored treatment with rosiglitazone (*P* = 0.01) and combination therapy (*P* = 0.03). Three individuals left the study, and two due to adverse events (one patient could not tolerate metformin and one stopped treatment due to hypertriglyceridemia).

### Patients with NAFLD (without diabetes)

Nine trials enrolled NAFLD patients without diabetes and included metformin [10, 15, 22, 23],} pioglitazone [10–13, 22],} liraglutide [9], rosiglitazone [17], and rosiglitazone with metformin [16].

#### Liraglutide

A small trial (*N* = 52) of liraglutide compared with placebo conducted in the UK in adults with NASH (LEAN trial) allowed enrollment of patients with diabetes if the diabetes was well-controlled and stable (33% had diabetes) [9]. More patients taking liraglutide had a resolution of NASH at 48 weeks than with placebo (39% vs. 9%, RR 4.3, 95% CI 1.0 to 17.7). There was no difference in change in NAFLD activity score (− 1.3 vs. − 0.8, *P* = 0.24), fibrosis score (− 0.2 vs. 0.2, *P* = 0.11), ALT (− 26.6 vs. 10.2 IU/L, *P* = 0.16), or AST (− 15.8 vs. − 8.6 IU/L, *P* = 0.29). However, significantly more patients on placebo had worsening fibrosis (36% vs. 9%, RR 0.2, 95% CI 0.1 to 1.0, *P* = 0.04). There was greater weight loss with liraglutide (− 5.3 kg vs. − 0.6 kg, RR − 4.39 kg, 95% CI − 7.19 to − 1.59 kg) and greater improvement in HbA1c (− 0.53% vs. 0%, RR − 0.48%, 95% CI − 0.91 to − 0.05%). There were no differences between treatments in study withdrawals due to adverse events, risk of serious adverse events, or risk of gastrointestinal disorders.

#### Metformin

One small trial (*N* = 48) conducted in Norway randomized patients with NAFLD to treatment with metformin 2500 mg (3000 mg if body weight > 90 kg) or placebo for 6 months [15]. Twenty-seven percent of patients had diabetes. There was no difference between groups at end of treatment in steatosis, NAFLD activity score, or fibrosis. However, weight loss was greater with metformin than placebo (− 4.4 kg vs. + 0.3 kg, *P* < 0.001) as was reduction in HbA1c (− 0.2% to + 0.1%, *P* = 0.001) [23].

A second good-quality trial assessed 3 months of metformin (500 mg/day) use among Iranian adults with probable NAFLD by liver sonography [22]. Compared with placebo (*n* = 30), there was no difference among patients receiving metformin (*n* = 30) in change in BMI (− 0.6 vs. −0.7; *P* = 0.91), though ALT (− 10.1 vs. − 0.6 IU/L) and AST (− 6.4 vs. − 0.9 IU/L) were significantly reduced among those taking metformin. No adverse events were reported.

#### Pioglitazone

Three trials randomized patients without diabetes to pioglitazone or placebo [12, 13, 22]. In the first RCT, 163 patients with NASH were randomized to 30-mg pioglitazone or to placebo for 96 weeks [13]. Most patients (87%) underwent end-of-study biopsy. More patients treated with pioglitazone compared with placebo experienced improvement in liver histology (34% vs. 19%, *P* = 0.04), steatosis (69% vs. 31%, *P* < 0.001), NAFLD score (− 1.9 vs. 10.5, *P* < 0.001), and resolution of NASH (47% vs. 21%, *P* = 0.001). Liver function tests, fasting serum glucose, and insulin resistance were also significantly improved with pioglitazone versus placebo. However, weight gain was increased with pioglitazone compared with placebo (+ 4.7 kg vs. + 0.7 kg, *P* < 0.001). There was no difference between groups in change in liver fibrosis. Twelve patients experienced serious adverse events, with fewer events in the pioglitazone group (2.5% vs. 12%, RR 0.21, 95% CI 0.05 to 0.92).

In the second RCT, 74 patients with NASH were randomized to 30 mg of pioglitazone or placebo for 12 months [12]. All received diet and exercise counseling that was reinforced each visit. Sixty-one participants (82%) had liver biopsy at the end of treatment. A reduction in liver fat was seen with pioglitazone and placebo from baseline with no difference between groups. Pioglitazone was associated with a reduction in liver fibrosis compared with placebo (*P* = 0.05). Pioglitazone was also associated with greater improvement in HbA1c compared with placebo (− 0.2% vs. + 0.1%, *P* = 0.01), as well as greater improvement in ALT (− 37.1 vs. − 6.9 IU/L, *P* = 0.009). However, as above, pioglitazone treatment was associated with increase in body weight versus placebo (+ 2.6 kg vs. − 3.5 kg, *P* = 0.02). Withdrawals due to adverse events (18%) were similar between groups.

A third good-quality trial compared pioglitazone (15 mg/day) with placebo (*n* = 30 in each group) among Iranian adults with probable NAFLD by liver sonography [22]. While BMI was not different between groups (− 0.6 vs. − 0.7 kg/m^2^; *P* = 0.46), ALT (− 8.6 vs. − 0.6 IU/L; *P* < 0.001) and AST (− 6.7 vs. − 0.9 IU/L; *P* < 0.001) were significantly reduced among those receiving pioglitazone. No adverse events were reported.

#### Metformin or pioglitazone

In a randomized trial conducted in India, 98 patients were allocated to metformin, rosuvastatin, or pioglitazone [10]. At 24 weeks, change in ultrasound fatty liver score significantly favored rosuvastatin and pioglitazone over metformin (− 1.27 vs. − 0.70 vs. + 0.07, *P* < 0.001). Change in BMI favored metformin and rosuvastatin over pioglitazone (− 1.75 vs. − 1.54 vs. − 0.15 kg/m^2^, *P* < 0.001), whereas change in AST favored pioglitazone and metformin over rosuvastatin (− 23.73 IU/L vs. − 14.07 IU/L vs. + 8.06, *P* = 0.012). Adverse events were not reported.

A second trial that randomized 80 NAFLD patients to treatment with metformin 1000 mg or pioglitazone 30 mg for 4 months was conducted in Iran [14]. Eight percent of patients had diabetes. Both metformin and pioglitazone decreased body weight, liver function tests, fasting plasma glucose, and liver fat from baseline with no differences between treatments. No participant left the study due to adverse events or required a medication dose adjustment.

#### Pioglitazone or pentoxifylline

One randomized trial (*N* = 60) conducted in India compared 6-month treatment with pioglitazone 30 mg to treatment with pentoxifylline 1200 mg in patients with biopsy-proven NASH [11]. All patients received diet and exercise counseling. AST and ALT were both improved from baseline with pioglitazone (*P* = 0.003, both comparisons) but were not different from improvements with pentoxifylline. Improvements in liver fat and fasting blood sugar were also improved from baseline with pioglitazone (*P* = 0.005; *P* = 0.02, respectively) but were also not significantly different from improvement with pentoxifylline. Although patients gained weight with pioglitazone, the increase (2 kg) was not statistically significant (*P* = 0.31). No participant left the study due to adverse events.

#### Rosiglitazone

One RCT randomized 63 patients with NASH to rosiglitazone (4 mg/day for 1 month then 8 mg/day for 11 months) or placebo for 12 months [17] and found a reduction in liver fat with rosiglitazone (47% vs. 16%, *P* = 0.01), along with increased normalization of transaminases (38% vs. 7%, *P* = 0.005) but an increase in weight (+ 1.5 kg vs. − 1.0 kg, *P* < 0.01). There were no differences between groups in NAFLD Activity Score or HbA1c levels between treatments, although surrogate markers of insulin sensitivity were improved with rosiglitazone. Three patients treated with rosiglitazone experienced painful, swollen legs requiring dose adjustment, or discontinuation of treatment.

#### Rosiglitazone and metformin

One RCT randomized patients with NASH to 4 mg of rosiglitazone or to 4 mg of rosiglitazone plus 1000 mg of metformin or to 4 mg of rosiglitazone plus 50 mg of losartan for 48 weeks [16]. Seventeen percent of participants screened positive for diabetes (HbA1c > 6.5%). The baseline NAFLD activity score was different between groups (highest in the rosiglitazone-alone group at 5.1 and lowest in the rosiglitazone plus metformin group at 4.1, *P* = 0.014). Liver fat and fibrosis stages were similar between groups. Post-treatment liver biopsies showed no differences between groups on changes in NAFLD score, steatosis, fibrosis, or resolution of NASH (46% of 26 patients treated with rosiglitazone versus 36% of 28 patients treated with rosiglitazone and metformin). Liver function tests were improved in all treatment groups as was fasting serum glucose and insulin levels. The addition of metformin did not significantly help with weight gain versus treatment with rosiglitazone alone (− 1.2 kg vs. + 0.9 kg, *P* = 0.051). Twelve patients, at the recommendation of their physicians, terminated the study due to 12 different adverse events, some likely unrelated to treatment (e.g., terminal cancer).

## Discussion

Management of NAFLD involves treating the liver disease itself as well as associated metabolic comorbidities, including diabetes, obesity, and hyperlipidemia. A recent clinical practice guideline from the American Association for the Study of Liver Diseases (AASLD) [24] recommended that pharmacologic treatment aimed primarily at improving liver disease be limited to patients with biopsy-proven NASH and fibrosis, since patients without fibrosis generally have a favorable prognosis. As such, first-line treatment for most patients should focus on lifestyle intervention or pharmacologic treatment targeting diagnosed diabetes, obesity, or dyslipidemia. The AASLD guidelines recommend weight loss via hypocaloric diet and increased exercise, with a target of at least 7–10% weight loss to improve the majority of the histopathological features of NAFLD. The group recommends against metformin or GLP-1 agonists and recommends pioglitazone (a thiazolidinedione) in patients with biopsy-proven NASH, regardless of diabetes status, but recommends against using pioglitazone in NAFLD patients without fibrosis.

Consistent with the AASLD recommendation against metformin use in NAFLD patients, studies of metformin found no difference from placebo in steatosis, fibrosis, NAFLD activity score, or resolution of NASH. While weight and glucose control were improved with metformin, treatment with metformin did not substantially impact liver disease.

Studies of pioglitazone in NASH patients found benefits in liver function, liver fat, and NASH resolution, though significant increases in weight may be cause for concern. A recent systematic review and network meta-analysis by Sridharan et al. was consistent with our findings [25]: pioglitazone was found to be associated with better response than standard care (odds ratio 3.8, 95% CI, 2.0 to 7.4). Evidence for other thiazolinediones was more limited and had somewhat mixed results, but findings were generally consistent with those for pioglitazone: liver fat and function and glucose measures improved, but weight also increased.

While the AASLD guidelines recommend against using GLP-1 agonists in NASH patients, we found some evidence that liraglutide improves liver fat, liver function, and HbA1c and is effective at resolving NASH and reducing weight. Exenatide performed less well but also resulted in significant reductions in liver fat and weight.

The strengths of our study include the use of systematic review processes to identify all relevant studies that meet pre-defined inclusion criteria, assessment of the internal validity (i.e., quality) of included studies, and overall evaluation of the strength of evidence using an established approach. Limitations of the present review include restriction to English-language publications and restriction to randomized trials, which may have limited generalizability to real-world populations. Larger studies with longer follow-up are needed to better quantify the long-term benefits and harms of diabetes medications to treat NAFLD, since the disease state itself, as well as many of the common metabolic conditions associated with NAFLD, are chronic conditions with long natural histories. Additionally, longer and larger studies may provide further information on clinical health outcomes and uncommon adverse effects.

## Conclusions

Consistent with existing clinical practice guidelines, which recommend lifestyle intervention and treatment for comorbidities related to fatty liver disease as first-line treatment, trial evidence supports the efficacy of some diabetes drugs (especially pioglitazone) in patients with NAFLD or NASH, though weight gain with some diabetes drugs may warrant caution. Larger trials are needed to better characterize the efficacy and harms of diabetes pharmacotherapy in these patients.

## Supplementary information

**Additional file 1.** Search strategies.

## Data Availability

All data generated or analyzed during this study are included in this published article (and its supplementary information files).

## Abbreviations

AASLD: American Association for the Study of Liver Diseases
AHRQ: Agency for Healthcare Research & Quality
ALT: Alanine aminotransferase
AST: Aspartate aminotransferase
CI: Confidence interval
DERP: Drug Effectiveness Review Project
FDA: Food and Drug Administration
HbA1c: Glycated hemoglobin
IPD: Individual patient data
NAFLD: Nonalcoholic fatty liver disease
NASH: Nonalcoholic steatohepatitis
RCT: Randomized controlled trial
RR: Relative risk
