# Supplementary material for: Diabetes drugs for nonalcoholic fatty liver disease: a systematic review
Source: Syst Rev. 2019 Nov 29;8:295. doi: 10.1186/s13643-019-1200-8 (PMC6884753; doi:10.1186/s13643-019-1200-8)
Supplement: Supplementary file 1 — Additional file 1. Search strategies. [file 13643_2019_1200_MOESM1_ESM.docx]

## Additional file 1. Search strategies

## MEDLINE (searches through September 2019)

*1. steatohepatitis.mp. or Fatty Liver/*

*2. non-alcoholic steatohepatitis.mp.*

*3. non-alcoholic fatty liver disease.mp.*

*4. 1 or 2 or 3*

*5. limit 4 to english language*

*6. limit 5 to humans*

*7. limit 6 to clinical trial, all*

## Cochrane Central Register of Controlled Trials (searches through 3^rd^ quarter, 2019)

*1. Non-alcoholic fatty liver disease.mp. or Fatty Liver/ or exp Non-alcoholic Fatty Liver Disease/*

*2. non-alcoholic steatohepatitis.mp.*

*3. 1 or 2*

*4. limit 3 to clinical trial, all*
